# Lysosomal degradation of PD-L1 is associated with immune-related adverse events during anti-PD-L1 immunotherapy in NSCLC patients

**DOI:** 10.3389/fphar.2024.1384733

**Published:** 2024-05-10

**Authors:** Takeru Kashiwada, Ryotaro Takano, Fumihiko Ando, Shoko Kuroda, Yoshishige Miyabe, Ryuji Owada, Akihiko Miyanaga, Tomoko Asatsuma-Okumura, Masaaki Hashiguchi, Yoshikazu Kanazawa, Hiroshi Yoshida, Masahiro Seike, Akihiko Gemma, Yoshiko Iwai

**Affiliations:** ^1^ Department of Pulmonary Medicine and Oncology, Nippon Medical School, Tokyo, Japan; ^2^ Department of Cell Biology, Institute for Advanced Medical Sciences, Nippon Medical School, Tokyo, Japan; ^3^ Department of Gastrointestinal and Hepato-Biliary-Pancreatic Surgery, Nippon Medical School, Tokyo, Japan; ^4^ Department of Immunology and Parasitology, St. Marianna University School of Medicine, Kawasaki, Japan

**Keywords:** soluble PD-L1, inflammation, macrophage, lysosomal degradation, immune-related adverse event

## Abstract

**Background:** Immune checkpoint inhibitors (ICIs) can induce immune-related adverse events (irAEs). Liquid biomarkers to predict irAE occurrence are urgently needed. We previously developed an ELISA system to specifically detect soluble PD-L1 (sPD-L1) with PD-1-binding capacity (bsPD-L1). Here, we investigated the relationship between sPD-L1 and bsPD-L1 in gastric cancer (GC) and non-small cell lung cancer (NSCLC) treated with PD-1/PD-L1 blockade and their association with irAEs.

**Methods:** We examined sPD-L1, bsPD-L1, matrix metalloproteinases (MMPs), and proinflammatory cytokine levels by ELISA in plasma samples from 117 GC patients prior to surgery and 72 NSCLC patients prior to and at 2 months after ICI treatment (anti-PD-1, n = 48; anti-PD-L1, n = 24). In mice treated with anti-PD-1/PD-L1 antibodies (Abs), sPD-L1 levels and localization of Abs were examined by ELISA and immunohistochemistry, respectively.

**Results:**sPD-L1 was detected with higher frequency in GC patients than in NSCLC patients, whereas bsPD-L1 was detected with similar frequencies in GC and NSCLC patients. sPD-L1 levels were correlated with IL-1α, IL-1β, TNF-α, and IL-6 levels, while bsPD-L1 levels were correlated with MMP13, MMP3, and IFN-γ levels. In NSCLC patients, anti-PD-L1, but not anti-PD-1, treatment increased sPD-L1, which was associated with irAE development, but not with clinical outcomes. In mice, trafficking of anti-PD-L1 Abs to lysosomes in F4/80^+^ macrophages resulted in sPD-L1 production, which was suppressed by treatment with lysosomal degradation inhibitor chloroquine and macrophage depletion.

**Conclusion:** Anti-PD-L1-mediated lysosomal degradation induces sPD-L1 production, which can serve as an indicator to predict irAE development during anti-PD-L1 treatment.

## Introduction

Immune checkpoint inhibitors (ICIs) targeting programmed cell death 1 (PD-1) and its ligand, PD-L1, can induce durable anti-tumor response, but can also cause immune-related adverse events (irAEs) (Martins et al., 2019; Hommes et al., 2020). irAEs affect almost any organs and are sometimes fatal. Additionally, the onset of irAEs is unpredictable. Therefore, biomarkers to predict irAE occurrence are urgently needed.

Our early studies demonstrated the inhibitory role of PD-1/PD-L1 signaling in anti-tumor immune response (Freeman et al., 2000; Iwai et al., 2002; Iwai et al., 2005; Okazaki et al., 2013). PD-L1 is constitutively expressed on endothelial cells and macrophages in peripheral tissues (Freeman et al., 2000). Proinflammatory cytokines such as tumor necrosis factor (TNF)-α and interferon (IFN)-γ induce PD-L1 expression in various cell types, including immune, parenchymal, and tumor cells (Freeman et al., 2000). Upon ligation with PD-L1, PD-1 inhibits T cell proliferation and effector functions (Freeman et al., 2000; Okazaki et al., 2013). Both anti-PD-1 and anti-PD-L1 monoclonal antibodies (mAbs) enhance the T cell response, but the difference in their mechanism of action is not fully understood.

In addition to the membrane-bounded form, PD-L1 also has a soluble form (sPD-L1) (Wan et al., 2006; Chen et al., 2011). Many studies have conflicting results on the function of sPD-L1 (Frigola et al., 2011; Rossille et al., 2014; Wang et al., 2015). sPD-L1 is produced via several mechanisms (Chen et al., 2011; Dezutter-Dambuyant et al., 2016; Zhou et al., 2017; Chen et al., 2018; Gong et al., 2019), including RNA splicing, exosomal secretion, and proteolytic cleavage by matrix metalloproteinases (MMPs). We previously developed an ELISA system to specifically detect PD-1-binding sPD-L1 (bsPD-L1) (Takeuchi et al., 2018), and found that bsPD-L1 can function as an endogenous PD-1 blocker (Ando et al., 2024). Plasma bsPD-L1 is strongly correlated with MMP13 levels. bsPD-L1 and MMP13 levels are associated with intra-tumoral T cell infiltration and loss of extracellular matrix integrity, respectively, in the tumor microenvironment. The levels of MMP13 and its activator, MMP3, change during ICI treatment. Furthermore, the combination of bsPD-L1 and MMPs may serve as a non-invasive tool to predict the efficacy of ICIs. However, the relationship between bsPD-L1 and sPD-L1, and their difference in clinical significance remain unknown.

In this study, we investigated plasma bsPD-L1 and sPD-L1 levels in GC and NSCLC patients treated with PD-1/PD-L1 blockade. We found that anti-PD-L1, but not anti-PD-1, treatment induced sPD-L1 production, which may serve as a non-invasive tool to predict irAEs.

## Materials and methods

### Patients and specimens

This study included 117 patients diagnosed with GC between 2017 and 2020 at the Department of Gastrointestinal and Hepato-Biliary-Pancreatic Surgery in Nippon Medical School Hospital, Japan. Blood samples were collected from patients before surgery.

The study also included 72 patients diagnosed with NSCLC between 2017 and 2019 at the Department of Pulmonary Medicine and Oncology in Nippon Medical School Hospital, Japan. Blood was collected from patients prior to and at 2 months after the initiation of checkpoint immunotherapy (nivolumab, n = 20; pembrolizumab, n = 28; atezolizumab, n = 16; durvalumab, n = 8). Nivolumab (3 mg/kg, every 2 weeks), pembrolizumab (200 mg/body, every 3 weeks), or atezolizumab (1,200 mg/body, every 3 weeks) was administered until disease progression or unacceptable adverse events in a clinical setting. Durvalumab (10 mg/kg, every 2 weeks) was administered after curative chemoradiotherapy.

RECIST v1.1 was used to assess the efficacy of immunotherapy. Patients with progressive disease (PD) who were not evaluable for response by RECIST were determined by the treating physician as PD. We assessed disease control [DC; complete response (CR) + partial response (PR) + stable disease (SD)] at 2 months.

Baseline clinical and demographic data were collected from patient medical records. The study protocols (B-2019-005, and 28-09-646) were reviewed and approved by the Ethics Committee of Nippon Medical School. All participants provided written informed consent. This study was conducted in accordance with the Declaration of Helsinki.

### Animal experiments

C57BL/6 (B6) mice were purchased from Oriental Yeast Co., Ltd. (Tokyo, Japan). Mice were intravenously injected with 100 μg of anti-PD-1 (clone: J43, Invitrogen, Waltham, MA, United States of America, #16-9985-85) or anti-PD-L1 (clone: MIH5, Invitrogen #16-5982-85) mAbs, and then blood was collected at several timepoints. To induce inflammation, mice were intraperitoneally injected with Lipopolysaccharide (LPS) (10 mg/kg, Sigma-Aldrich, St. Louis, MO, United States of America, #L6511). To inhibit lysosomal degradation, mice were intraperitoneally injected with chloroquine (100 mg/kg/day, Sigma-Aldrich, #C6628) for 3 days prior to administration of Ab or LPS. To deplete macrophages, mice were intravenously injected with clodronate-containing or control liposomes (12.5 mg/kg, Hygieia Bioscience, Osaka, Japan, #16001003) at 24 h prior to Ab administration. All mice were maintained at the animal facility of Nippon Medical School. All animal experiments were approved by the Animal Care and Use Committee of Nippon Medical School (2020-018).

### Enzyme-linked immunosorbent assay (ELISA)

Human sPD-L1 and bsPD-L1 concentrations were measured as described previously (Takeuchi et al., 2018). Human MMP3, MMP9, MMP13, IL-1α, IL-1β, IL-6, TNF-α, and IFN-γ, and mouse sPD-L1 concentrations were determined using ELISA kits (R&D Systems, Minneapolis, MN, United States of America, #DY513, #DY911, #DY511, #DY200, #DY201, #DY206, #DY210, #DY285B, and #DY1019, respectively), in accordance with the manufacturer’s instructions.

### Immunohistochemistry

Mice were intravenously injected with 100 μg of Alexa 488-labeled mAb, and then organs were harvested 24 h later. Cryosections were fixed and permeabilized by Cytofix/Cytoperm solution (BD Biosciences, Franklin Lakes, NJ, United States of America, #554722) and stained with anti-B220-PE (BioLegend, San Diego, CA, United States of America, #103208) and anti-F4/80-Alexa Fluor 647 (BioLegend, #123122) mAbs. To examine subcellular localization of mAbs, cryosections were stained with anti-Rab7 (Abcam, Cambridge, UK, #ab137029) or anti-LAMP1 (Abcam #ab24170) Abs in combination with anti-F4/80-Alexa Fluor 647, followed by anti-rabbit IgG-Cy3 (Jackson ImmunoResearch, West Grove, PA, United States of America, #711-166-152). The sections were mounted with Prolong Gold with DAPI (Invitrogen, #P36931) and analyzed by confocal microscopy (Olympus, Tokyo, Japan, #FV1200). Images were processed using Imaris software, version 9.7.1(Oxford Instruments, Abingdon, UK).

### Statistical analysis

Mann-Whitney U test or Student’s t-test was used to compare numerical variables, and Pearson’s chi-squared test was used to compare categorical variables. Receiver operator characteristic (ROC) curve, along with area under the ROC (AUC), was used to assess the discriminatory ability of the numerical variables. All tests were two-tailed, and *p*-values <0.05 were considered statistically significant. All statistical analyses were performed using JMP software, version 13 (SAS Institute, Cary, NC, United States of America) and Prism software, version 8 (GraphPad, San Diego, CA, United States of America).

## Results

### Detection of sPD-L1 with higher frequency in GC patients than in NSCLC patients

**FIGURE 1:**
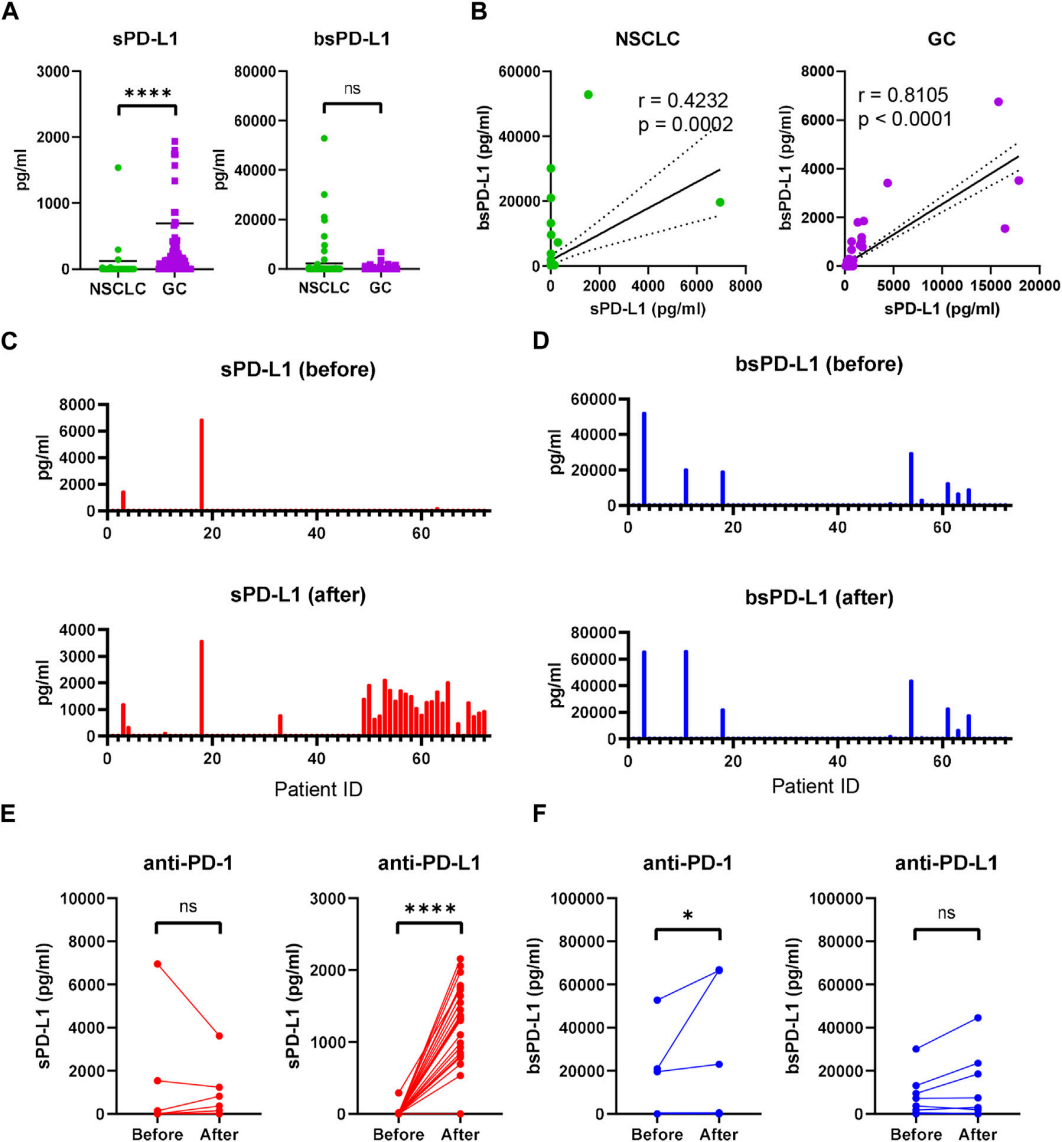
Detection of sPD-L1 and bsPD-L1 in the plasma of GC and NSCLC patients. **(A)** Baseline sPD-L1 and bsPD-L1 levels in plasma samples from GC patients (n = 117) and NSCLC patients (n = 72). **(B)** Correlation between baseline sPD-L1 and bsPD-L1 levels in GC and NSCLC patients. r indicates the correlation coefficient. **(C)** sPD-L1 levels and **(D)** bsPD-L1 levels in plasma samples from NSCLC patients prior to and at 2 months after ICI treatment. **(E)** Kinetic change of sPD-L1 and **(F)** bsPD-L1 levels in NSCLC patients prior to and at 2 months after anti-PD-1 (n = 48) or anti-PD-L1 (n = 24) treatment. r indicates the correlation coefficient. Horizontal lines indicate the mean. Statistical significance was calculated using the Mann–Whitney U test **(A, E, and F)**. *p < 0.05; ****p < 0.0001; ns, not significant.

We examined baseline sPD-L1 and bsPD-L1 levels in plasma samples from 117 GC patients and 72 NSCLC patients (Figure 1A). sPD-L1 was detected with higher frequency in GC patients (90/117, 76.9%) than in NSCLC patients (6/72, 8.3%), whereas bsPD-L1 was detected with similar frequencies in GC patients (17/117, 14.5%) and NSCLC patients (17/72, 22.2%). The average sPD-L1 level in GC patients was higher than that in NSCLC patients (693 ± 2668 and 125 ± 837 pg/mL, respectively), while the average bsPD-L1 level in NSCLC patients was higher than that in GC patients (2237 ± 7,960 and 216 ± 823 pg/mL, respectively). We found a stronger correlation between sPD-L1 and bsPD-L1 levels in GC patients than in NSCLC patients (correlation coefficient [r] = 0.8105 and r = 0.4232, respectively; Figure 1B). These results suggest different expression patterns of sPD-L1 and bsPD-L1 between GC and NSCLC.

### Increased sPD-L1 levels during anti-PD-L1 treatment in NSCLC patients

Next, we investigated kinetic changes of sPD-L1 and bsPD-L1 in NSCLC patients during ICI treatment. Pretreatment patient characteristics are summarized in Supplementary Table S1. No significant differences were found in any of the variables between anti-PD-1- and anti-PD-L1-treated groups. At 2 months of treatment, sPD-L1^+^ patients were increased (Figure 1C), while bsPD-L1^+^ patients were unchanged (Figure 1D). Subgroup analysis revealed that anti-PD-L1 treatment increased sPD-L1 levels, but not bsPD-L1 levels (Figures 1E,F).

### Correlation between sPD-L1 and proinflammatory cytokine levels

**FIGURE 2:**
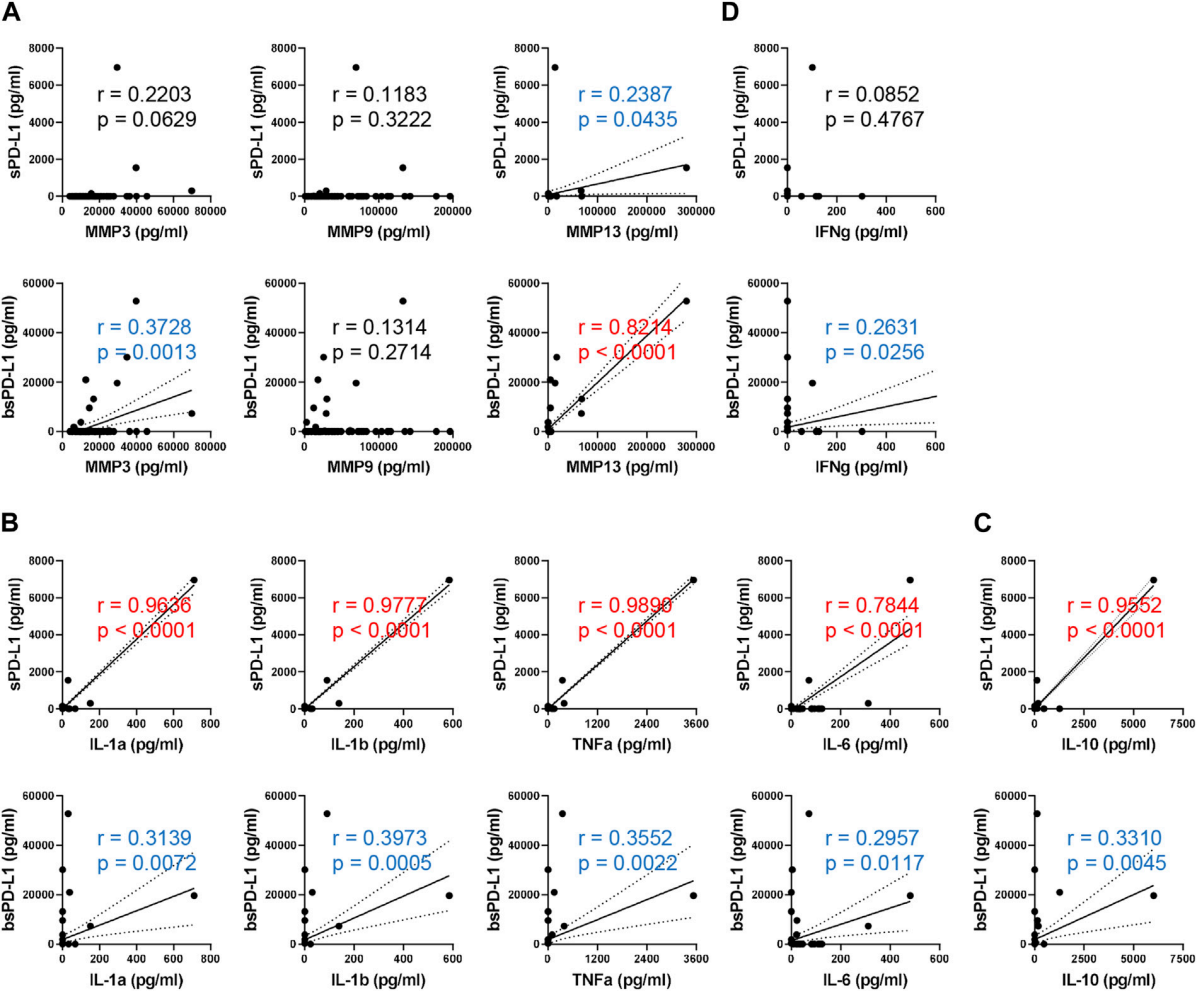
Correlation between baseline sPD-L1 and proinflammatory cytokine levels in NSCLC patients. **(A)** Correlation between baseline MMPs (MMP3, MMP9, and MMP13) and sPD-L1 or bsPD-L1 levels in NSCLC patients (n = 72). **(B)** Correlation between baseline proinflammatory cytokines (IL-1α, IL-1β, TNF-α, and IL-6) and sPD-L1 or bsPD-L1 levels in NSCLC patients (n = 72). **(C)** Correlation between baseline IL-10 and sPD-L1 or bsPD-L1 levels in NSCLC patients (n = 72). **(D)** Correlation between baseline IFN-γ and sPD-L1 or bsPD-L1 levels in NSCLC patients (n = 72). r indicates the correlation coefficient.

It has been reported that PD-L1 is selectively cleaved by MMP13 and MMP9, *in vitro* (Dezutter-Dambuyant et al., 2016). We investigated the relationship between baseline MMPs and sPD-L1 or bsPD-L1 in NSCLC patients (Figure 2A). MMP13 levels were strongly corelated with bsPD-L1 levels, but weakly correlated with sPD-L1 levels. MMP3 levels were moderately correlated with bsPD-L1, but not with sPD-L1. MMP9 levels were not correlated with either sPD-L1 or bsPD-L1. These results suggest that MMP13 may be involved in the generation of bsPD-L1 rather than sPD-L1.

We investigated the relationship between proinflammatory cytokines and sPD-L1 or bsPD-L1 levels (Figure 2B). sPD-L1 levels were strongly correlated with IL-1α, IL-1β, TNF-α, and IL-6, whereas bsPD-L1 levels were weakly correlated with these cytokines, suggesting that inflammation may be involved in the generation of sPD-L1 rather than bsPD-L1. Furthermore, IL-10, an anti-inflammatory cytokine, showed a strong or weak correlation with sPD-L1 or bsPD-L1 levels, respectively (Figure 2C). On the other hand, IFN-γ levels were weakly correlated with bsPD-L1 levels (r = 0.2631), but not with sPD-L1 levels (Figure 2D), suggesting that bsPD-L1, but not sPD-L1, may regulate T cell response.

**FIGURE 3:**
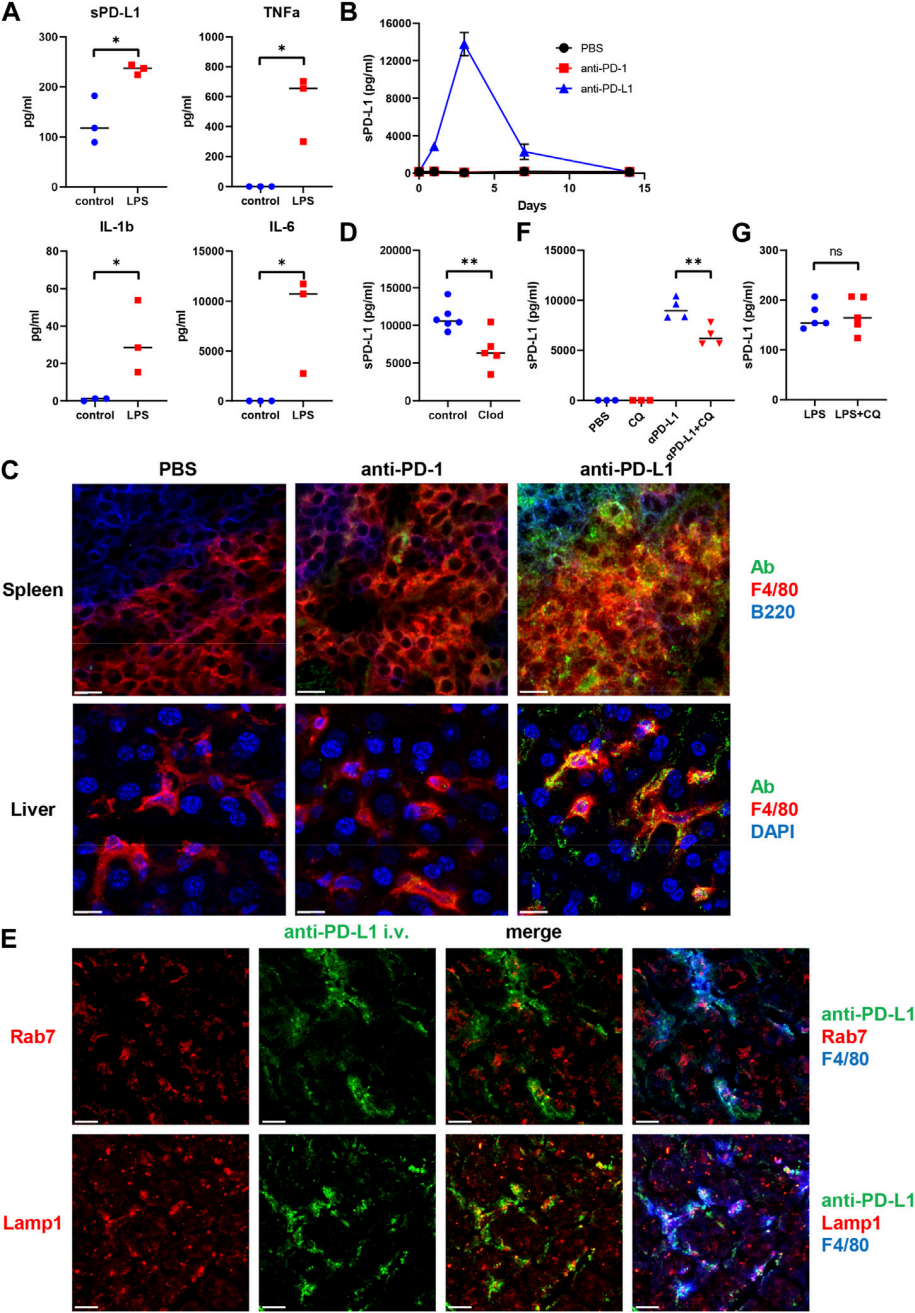
Intracellular trafficking of anti-PD-L1 mAb in mouse F4/80^+^ macrophages. **(A)** Plasma sPD-L1 and proinflammatory cytokine levels in mice after 2 h of treatment with Lipopolysaccharide (LPS). **(B)** Plasma sPD-L1 levels in mice at the indicated time points after administration of PBS, anti-PD-1, or anti-PD-L1 mAb. **(C)** Mice were injected with PBS, Alexa 488-labeled anti-PD-1, or anti-PD-L1 mAb (green), and then organs were harvested 24 h later. Spleen sections were stained with anti-F4/80 (red) and anti-B220 (blue) mAbs. Liver sections were stained with anti-F4/80 mAb (red) and DAPI (blue). Scale bars indicate 20 µm. **(D)** Mice were treated with clodronate-containing or control liposomes and then injected with anti-PD-L1 mAb. Blood samples were collected 24 h later, and sPD-L1 levels were analyzed by ELISA. **(E)** Mice were injected with Alexa 488-labeled anti-PD-L1 mAb (green), and then organs were harvested 24 h later. Liver sections were stained with anti-Rab7 or anti-LAMP1 Ab (red) in combination with anti-F4/80 mAb (blue). Scale bars indicate 20 µm. **(F)** Mice were treated with chloroquine (CQ) for 3 days and then injected with anti-PD-L1 mAb. Blood samples were collected 24 h later and sPD-L1 levels were analyzed by ELISA. **(G)** Mice were treated with CQ for 3 days and then injected with LPS. Blood samples were collected 2 h later and sPD-L1 levels were analyzed by ELISA. Horizontal lines indicate the mean. Statistical significance was calculated using the Student’s t-test **(A, D, F, and G)**. *p < 0.05; **p < 0.01; ns, not significant.

To examine whether inflammation might induce sPD-L1 production, mice were intraperitoneally injected with LPS. As a result, LPS treatment increased the levels of sPD-L1 as well as proinflammatory cytokines such as IL-1β, TNF-α, and IL-6 (Figure 3A), suggesting that inflammation may be involved in the production of sPD-L1.

### Internalization of anti-PD-L1 mAb by mouse F4/80^+^ macrophages

To examine the mechanism of sPD-L1 production during immunotherapy, mice were treated with anti-PD-1 or anti-PD-L1 mAb (Figure 3B). Consistent with NSCLC patients, sPD-L1 levels were increased in mice treated with anti-PD-L1 mAb, but not with anti-PD-1 mAb. sPD-L1 levels began to increase at day 1, reached a peak at day 3 and remained detectable at day 7 of anti-PD-L1 mAb administration.

To investigate the tissue distribution of anti-PD-L1 mAb *in vivo*, mice were treated with fluorescently labeled anti-PD-L1 mAb. Splenic F4/80^+^ macrophages accumulated intracellular fluorescence (Figure 3C). The anti-PD-L1 mAb was localized predominantly inside the cells, with little remaining on the cell surface. However, the fluorescently labeled anti-PD-1 mAb remained almost exclusively on the surface of splenic F4/80^+^macrophages. The internalization of anti-PD-L1 mAb with punctate staining was also observed in liver and bone marrow F4/80^+^ macrophages (Figure 3C; Supplementary Figure S1). These results suggest that the injected anti-PD-L1 mAb was internalized by macrophages in various tissues.

### Macrophages as the source of sPD-L1 during anti-PD-L1 treatment

To examine whether macrophages might be involved in the Ab-mediated sPD-L1 production, mice were treated with clodronate-containing liposomes to delete macrophages prior to the anti-PD-L1 mAb administration. sPD-L1 levels were lower in mice treated with clodronate-containing liposomes than in those treated with control liposomes (Figure 3D), suggesting that macrophages are the source of sPD-L1 during anti-PD-L1 treatment.

To investigate the subcellular localization of anti-PD-L1 mAb, mice were injected with the fluorescently labeled anti-PD-L1 mAb. After 24 h, liver sections were stained with anti-Rab7 or anti-Lamp1 Ab (Figure 3E). Rab7 and Lamp1 are markers of late endosome and lysosome, respectively. Anti-PD-L1 staining was highly punctate in F4/80^+^macrophages. Some co-localization was observed between anti-PD-L1 and anti-Lamp1 Abs, suggesting that the anti-PD-L1 mAb may traffic to lysosomes in F4/80^+^ macrophages after internalization.

Ab binding to cell surface molecules often triggers endocytosis of antigen/Ab complexes, which are delivered to lysosomes for degradation (Zhang et al., 2019). To examine whether lysosomal degradation might be involved in sPD-L1 production, mice were treated with chloroquine prior to the anti-PD-L1 mAb administration. sPD-L1 levels were significantly decreased in chloroquine-treated mice compared with untreated mice (Figure 3F). Conversely, chloroquine treatment did not suppress LPS-induced sPD-L1 production (Figure 3G). These results suggest that lysosomal degradation plays an important role in Ab-mediated sPD-L1 production, but not in LPS-induced sPD-L1 production.

### Elevated sPD-L1 in NSCLC patients with irAEs during anti-PD-L1 treatment

**FIGURE 4:**
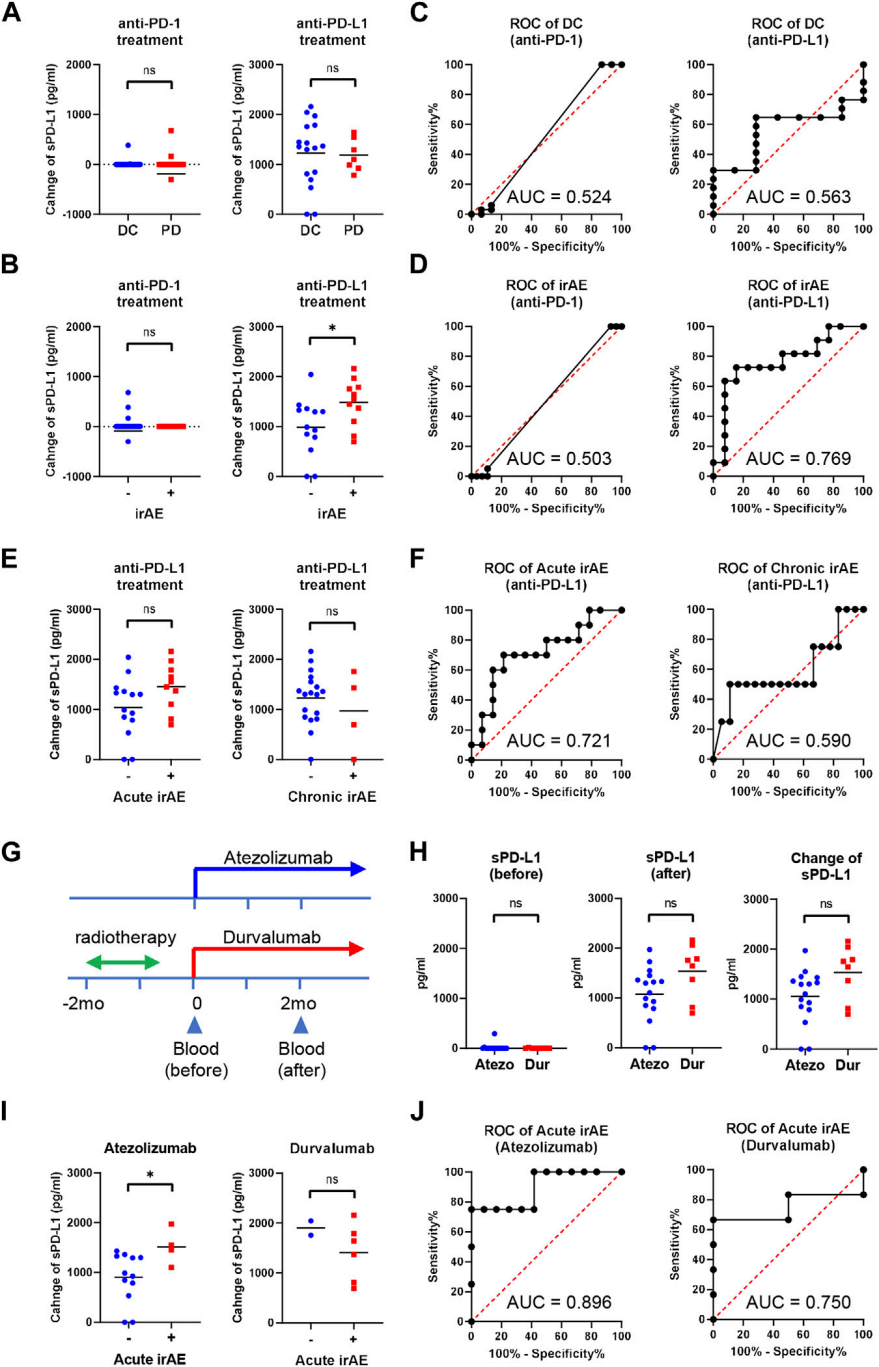
Increased sPD-L1 levels in NSCLC patients with irAEs during anti-PD-L1 treatment. **(A)** Comparison of the sPD-L1 change between patients with disease control (DC, n = 33) and progressive disease (PD, n = 15) at 2 months of anti-PD-1 treatment (left), or between patients with DC (n = 17) and PD (n = 7) at 2 months of anti-PD-L1 treatment (right). **(B)** Comparison of the sPD-L1 change between patients with irAEs (n = 20) and without irAEs (n = 28) during anti-PD-1 treatment (left), or between patients with irAEs (n = 11) and without irAEs (n = 13) during anti-PD-L1 treatment (right). **(C)** ROC curve analysis of the sPD-L1 change to predict DC in patients with anti-PD-1 or anti-PD-L1 treatment. **(D)** ROC curve analysis of the sPD-L1 change to predict irAEs in patients with anti-PD-1 or anti-PD-L1 treatment. **(E)** Comparison of the sPD-L1 change between patients with acute irAEs (n = 10) and without acute irAEs (n = 14) during anti-PD-L1 treatment (left), or between patients with chronic irAEs (n = 4) and without chronic irAEs (n = 20) during anti-PD-L1 treatment (right). **(F)** ROC curve analysis of the sPD-L1 change to predict acute or chronic irAEs in patients with anti-PD-L1 treatment. **(G)** A schematic diagram of anti-PD-L1 treatment. **(H)** sPD-L1 levels prior to (left) and 2 months after (middle) treatment with atezolizumab (n = 16) or durvalumab (n = 8). sPD-L1 change (right) in patients during treatment with atezolizumab (n = 16) or durvalumab (n = 8). **(I)** Comparison of the sPD-L1 change between patients with acute irAEs (n = 4) and without acute irAEs (n = 12) during atezolizumab treatment (left), or between patients with acute irAEs (n = 6) and without acute irAEs (n = 2) during durvalumab treatment (right). **(J)** ROC curve analysis of the sPD-L1 change to predict acute irAEs in patients treated with atezolizumab or durvalumab. The Horizontal lines indicate the mean. Statistical significance was calculated using the Student’s t-test **(A, B, E, H, and I)**. *p < 0.05; ns, not significant.

We investigated whether the sPD-L1 change was associated with clinical response to ICIs in NSCLC patients (Figure 4A). At 2 months of treatment, 33 of 48 anti-PD-1-treated patients and 17 of 24 anti-PD-L1-treated patients obtained DC. No significant difference was found in the sPD-L1 change between patients with DC and PD in both treatment groups. We also examined the association of the sPD-L1 change with irAE development during ICI treatment (Figure 4B). After ICI treatment, 20 of 48 anti-PD-1-treated patients and 11 of 24 anti-PD-L1-treated patients developed irAEs. Patients with irAEs experienced significantly greater increase in sPD-L1 than patients without irAEs during anti-PD-L1 treatment (*p* = 0.0321), but not during anti-PD-1 treatment.

We performed ROC analysis to assess the discriminatory ability of the sPD-L1 change during anti-PD-L1 treatment. The AUC of the sPD-L1 change to predict DC was 0.563, indicating a low discriminatory ability of change in sPD-L1 for clinical outcomes (Figure 4C). Conversely, the AUC of the sPD-L1 change to predict irAE was 0.769, suggesting a relatively high discriminatory ability (Figure 4D). These results suggest the potential utility of sPD-L1 change to predict irAEs during anti-PD-L1 treatment. Of the 24 patients treated with anti-PD-L1, 10 developed irAEs during the acute phase (within the first 2 months of treatment), and 4 during the chronic phase (after 2 months of treatment) (Figure 4E). The AUC of the sPD-L1 changes predicting irAE in the acute and chronic phases were 0.721 and 0.590, respectively (Figure 4F).

The anti-PD-L1-treated patients were then subdivided into atezolizumab-treated (without radiotherapy) and durvalumab-treated (with radiotherapy) groups (Figure 4G). There was a trend toward greater increases in sPD-L1 in the durvalumab-treated group compared to the atezolizumab-treated group, but the difference was insignificant (*p* = 0.0511, Figure 4H). Within 2 months of treatment, acute irAE occurred in 4 of 16 atezolizumab-treated patients and 6 of 8 durvalumab-treated patients (Figure 4I). Patients with acute irAEs had significantly greater increase in sPD-L1 than those without acute irAEs during atezolizumab treatment (*p* = 0.0407), but not during durvalumab treatment. The AUC of the sPD-L1 change to predict acute irAE during atezolizumab treatment was 0.896 (Figure 4J), indicating a high discriminatory ability.

## Discussion

In this study, we investigated the relationship between sPD-L1 and bsPD-L1 in GC and NSCLC patients. sPD-L1 was detected with higher frequency in GC patients than in NSCLC patients, whereas bsPD-L1 was detected with similar frequencies in GC and NSCLC patients. In NSCLC patients, bsPD-L1 levels were almost unchanged during ICI treatment (Figures 1C, D), whereas anti-PD-L1, but not anti-PD-1, treatment increased sPD-L1 levels (Figure 1E). The sPD-L1 increase was associated with irAE development, but not with clinical outcomes (Figure 4). These results suggest that the sPD-L1 change can serve as an indicator to predict irAEs during anti-PD-L1 treatment.

Our results revealed the mechanism and source of sPD-L1 production during anti-PD-L1 treatment. Consistent with NSCLC patients, sPD-L1 levels were increased in mice treated with anti-PD-L1 mAb, but not with anti-PD-1 mAb. We observed trafficking of the anti-PD-L1 mAb to lysosomes in F4/80^+^ macrophages in various tissues (Figures 3C, E). Treatment with chloroquine inhibited the sPD-L1 increase induced by the anti-PD-L1 mAb, suggesting the involvement of lysosomal digestion in sPD-L1 production (Figure 3F). Furthermore, depletion of macrophages by clodronate-containing liposomes suppressed the anti-PD-L1-induced sPD-L1 increase (Figure 3D), suggesting that macrophages are the major source of sPD-L1 during anti-PD-L1 treatment. Our findings suggest that the mechanism of anti-tumor effect by anti-PD-L1 mAb is more likely to be degradation of PD-L1 rather than blockade of PD-1/PD-L1 signaling.

We also found that sPD-L1 production might be influenced by the tumor location. sPD-L1 was detected with much higher frequency in GC patients than in NSCLC patients (Figure 1A). We observed a stronger correlation between sPD-L1 and bsPD-L1 levels in GC patients than in NSCLC patients (Figure 1B). Because the gastrointestinal tract contains many digestive enzymes compared with lung tissues, it is likely that bsPD-L1 degradation by digestive enzymes contributes to sPD-L1 production.

bsPD-L1 levels were correlated with MMP13, MMP3, and IFN-γ levels (Figure 2A, D), while sPD-L1 levels were correlated with IL-1α, IL-1β, TNF-α, and IL-6 levels (Figure 2B). We speculate that MMP-mediated cleavage might be involved in bsPD-L1 production, whereas inflammation might be involved in sPD-L1 production. We have previously reported that glycosylation of bsPD-L1 is important for its binding to PD-1 (Takeuchi et al., 2018). MMP-mediated PD-L1 cleavage may retain its conformational structure and glycosylated sites necessary for PD-1 binding. We found that sPD-L1 levels were increased in LPS-induced inflammation model, but not suppressed by chloroquine treatment (Figure 3A, G). These results suggest that mechanisms other than lysosomal degradation may be involved in LPS-induced sPD-L1 production. We also note that durvalumab-treated patients showed a trend toward a greater increase in sPD-L1 than atezolizumab-treated patients (Figure 4H). Since radiation therapy precedes durvalumab treatment, radiation-induced inflammation may enhance sPD-L1 production.

In this study, we demonstrated the association of sPD-L1 increase with irAE development, but not with clinical outcomes during anti-PD-L1 treatment (Figure 4). We have recently demonstrated that bsPD-L1 can function as an endogenous PD-1 blocker and serve as a biomarker for predicting the efficacy of ICIs (Ando et al., 2024). Unlike bsPD-L1, sPD-L1 without PD-1-binding ability does not have immunological functions to enhance T cell response. However, sPD-L1 production might reflect PD-L1 degradation in peripheral tissues, which causes a breakdown of peripheral tolerance leading to irAE development. Thus, the sPD-L1 change during anti-PD-L1 treatment may serves as an indicator to predict irAEs. Indeed, sPD-L1 change showed a high discriminatory ability to predict acute irAE development during atezolizumab treatment (Figure 4J). Considering the results that sPD-L1 levels reached a peak at day 3 of anti-PD-L1 administration in the mouse model, the time interval to assess sPD-L1 changes should be shortened for early diagnostics. Since sPD-L1 and bsPD-L1 have different clinical values, the combination of sPD-L1 and bsPD-L1 might represent a powerful non-invasive diagnostic tool to improve cancer immunotherapy by preventing side effects and stratifying patients.

## Data Availability

The original contributions presented in the study are included in the article/Supplementary Material, further inquiries can be directed to the corresponding authors.
