# Supplementary material for: Lysosomal degradation of PD-L1 is associated with immune-related adverse events during anti-PD-L1 immunotherapy in NSCLC patients
Source: Front Pharmacol. 2024 May 10;15:1384733. doi: 10.3389/fphar.2024.1384733 (PMC11116720; doi:10.3389/fphar.2024.1384733)
Supplement: Supplementary file 1 [file Table1.pdf]

Supplementary Table 1 Characteristics of NSCLC patients

Variable		All patients (N=72)	anti-PD-1 (N=48)	anti-PD-L1 (N=24)	p value
ICI treatment					
	Nivolumab	20 (27.8%)	20 (41.6%)		
	Pembrolizumab	28 (38.9%)	28 (58.3%)		
	Atezolizumab	16 (22.2%)		16 (66.7%)	
	Durvalumab	8 (11.1%)		8 (33.3%)	
Age	Median (range)	67 (28-88)	70 (28-88)	62 (43-77)	0.0551
Gender					0.4227
	Male	56 (77.8%)	36 (75.0%)	20 (83.3%)	
	Female	16 (22.2%)	12 (25.0%)	4 (16.7%)	
Smoking					0.3542
	Never	11 (15.3%)	6 (12.5%)	5 (20.8%)	
	Current or Former	61 (84.7%)	42 (87.5%)	19 (79.2%)	
EGFR Mutation					0.7706
	Wild	60 (88.2%)	42 (87.5%)	18 (90.0%)	
	Mutant	8 (11.8%)	6 (12.5%)	2 (10.0%)	
T					0.2482
	-1c	18 (25.0%)	14 (29.2%)	4 (16.7%)	
	2a-	54 (75.0%)	34 (70.8%)	20 (83.3%)	
N					0.2468
	0	11 (15.3%)	9 (18.8%)	2 (8.3%)	
	1-	61 (84.7%)	39 (81.2%)	22 (91.7%)	
M					0.1528
	0	49 (68.1%)	30 (62.5%)	19 (79.2%)	
	1	23 (31.9%)	18 (37.5%)	5 (20.8%)	
Stage					0.0916
	-II	10 (13.9%)	9 (18.8%)	1 (4.2%)	
	III-	62 (86.1%)	39 (81.2%)	23 (95.8%)	
Pathology					0.2451
	Squamous cell carcinoma	32 (44.4%)	24 (50.0%)	8 (33.3%)	
	Adenocarcinoma	32 (44.4%)	18 (37.5%)	14 (58.3%)	
	Non-small	8 (11.2%)	6 (12.5%)	2 (8.4%)	
PD-L1 TPS status					0.1500
	<50	31 (52.5%)	19 (46.3%)	12 (66.7%)	
	50≤	28 (47.5%)	22 (53.7%)	6 (33.3%)	
