# Supplementary figures and images for: Lysosomal degradation of PD-L1 is associated with immune-related adverse events during anti-PD-L1 immunotherapy in NSCLC patients
Source: Front Pharmacol. 2024 May 10;15:1384733. doi: 10.3389/fphar.2024.1384733 (PMC11116720; doi:10.3389/fphar.2024.1384733)

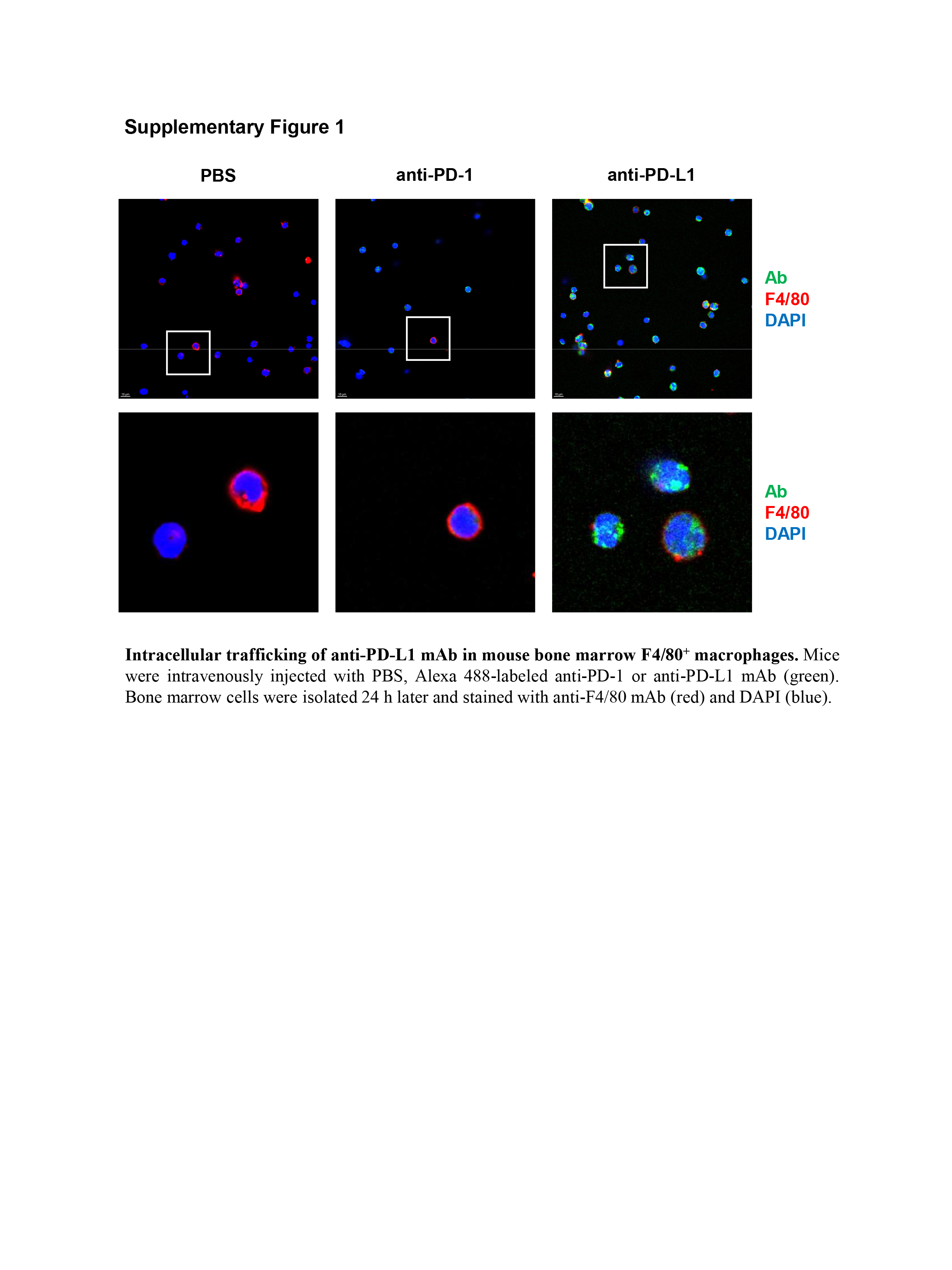

Supplement: Supplementary file 2 [file Image1.TIF]
